# Self-regulation in chemical and bio-engineering materials for intelligent systems

**DOI:** 10.1049/trit.2018.0004

**Published:** 2018-04-04

**Authors:** Zhongyuan Huang, Kewei Lei, Dan He, Yanbin Xu, Jacob Williams, Liu Hu, Macy McNeil, Juan M. Ruso, Zhen Liu, Zhanhu Guo, Zhe Wang

**Affiliations:** 1 Chemistry Department, Xavier University of Louisiana, New Orleans, LA 70125, USA; 2 College of Chemistry and Chemical Engineering, Xinyang Normal University, Xinyang 464000, Henan, People’s Republic of China; 3 Materials Science and Chemical Engineering, Ningbo University, Ningbo 315211, People’s Republic of China; 4 Department of Pharmaceutical Analysis, Chongqing Medical University, Chongqing 400016, People’s Republic of China; 5 School of Chemistry and Materials Science, Ludong University, Yantai 264025, Shandong, People’s Republic of China; 6 Department of Physics and Engineering, Frostburg State University, Frostburg, MD 21532, USA; 7 Department of Mechanical Engineering, University of Maryland, College Park, MD 20742, USA; 8 Integrated Composites Laboratory (ICL), Department of Chemical & Biomolecular Engineering, University of Tennessee, Knoxville, TN 37996, USA; 9 National Engineering Research Center for Advanced Polymer Processing Technology, Zhengzhou University, Zhengzhou 450002, People’s Republic of China; 10 Soft Matter and Molecular Biophysics Group, Department of Applied Physics, University of Santiago de Compostela, 15782 Santiago de Compostela, Spain

## Abstract

Herein, the authors review the self-regulation system secured by well-designed hybrid materials, composites, and complex system. As a broad concept, the self-regulated material/system has been defined in a wide research field and proven to be of great interest for use in a biomedical system, mechanical system, physical system, as the fact of something such as an organisation regulating itself without intervention from external perturbation. Here, they focus on the most recent discoveries of self-regulation phenomenon and progress in utilising the self-regulation design. This paper concludes by examining various practical applications of the remarkable materials and systems including manipulation of the oil/water interface, cell out-layer structure, radical activity, electron energy level, and mechanical structure of nanomaterials. From material science to bioengineering, self-regulation proves to be not only viable, but increasingly useful in many applications. As part of intelligent engineering, self-regulatory materials are expected to be more used as integrated intelligent components.

## 1 Introduction

Various methods have been used to regulate characteristics of materials in both the chemical and chemical engineering industries in recent years [1–6], which regulate the hydrophobic [7–10], antibiotic [11–15], radical polymerisation [16–20], electrical conductivity [21–23], thermal conductivity [23, 24], and mechanochemical properties [25–31] within porous, biological, polymer, and photoelectric materials. The materials, which have the ability to regulate above characteristics, could be allowed to be utilised in many different and innovative applications [32–34]. Moreover, these applications will have very positive impacts and change their respective industries [35–38].

Taking porous materials as an example, their structure could be altered so that the materials can accept and filter substances from liquid [39–41]. Typically, porous hydrophobic/oleophilic material (PHOM), the original polymer sponges (PSs), has the potential to change the environmental engineering industry [42]. Coating with a hydrophobic material, the polymers will become hydrophobic PSs (HPSs) and can be used for particular applications. By using PHOMs along with applied suction, oil may be cleaned out of the water more quickly and efficiently through capillary action.

In medical apparatuses and instruments field, self-regulation is also found in certain polymers that are used to clean medical devices [43–45]. Depending on the bacteria in the surrounding, these polymers adjust and release disinfectant. By using the disinfectant released by this kind of polymers, people can regulate how much antibiotic is released on a site, and know the level of antibiotic resistance. It is not only applicable to medical instruments, but also to the cleaning of actual wounds. The polymer will only release antibiotic when surrounded by certain bacteria, so it can be accepted better if they are incorporated into wound dressing and among other things. Moreover, self-regulation is common in the controlled radical polymerisation. Through a new approach and controlled radical polymerisation, monomers can be polymerised without spontaneous initiation.

Self-regulation is found in the regulation of charge in, especially transition-metal (TM) compounds [46–48]. The charge of photoelectric materials may be regulated through various oxidation and reduction processes, which can regulate the concentration of charge carriers and then defect formation, and affect the photovoltaic properties of the material.

Moreover, self-regulation may be proved to be particularly useful in the biological field [49–51]. It is well known that organisms can adjust to their surroundings through chemo-mechanochemical feedback loops, in which regulation is widely existing [52–54]. Through self-regulated mechanochemical adaptively reconfigurable tunable system (SMARTS), we can study the chemomechanical events, and give us the way to know the regulation of chemical reactions due to mechanical actions. This can be applied to various regulating processes such as pH levels, heat, light, glucose formation, and so on. A particular application is using a material composed of microstructures to form a reaction that can be turned on or off. When the catalyst is coated on the tips of the microstructures in a liquid bilayer of nutrient and reactant layer, the system switches on or off with the microstructures being straight or bent.

Clearly, self-regulation can be applied in various fields and has been shown to be very versatile in its application. Moreover, it has the potential to change the fields, in which it has been applied, and it is already having a positive impact on those fields. From material engineering to chemical engineering industries, self-regulation proves to be not only viable, but also increasingly useful in many applications. In view of the artificial intelligence operation mode, we can forecast intelligent computing and intelligent control will be an important part of the future intelligent engineering. The intelligent materials with automatic control performance would be accurate to accept and regulate the work instructions of the intelligent distributed computing centre.

In the following sections (from Sections 2 to 7), we will review the six major research areas which directly related to the self-regulation behaviours in chemical and bio-engineering materials for intelligent systems, mainly focus on the porous materials, biomaterials, polymeric materials, photoelectric materials, mechanochemical systems, and energy materials. In conclusions and perspectives part, our review will not only summarise the current research highlights, but also offer the research perspective to project the future development and designing the self-regulation system and their interactions throughout interdisciplinary subjects.

## 2 Physical self-regulation based on porous materials

Porous materials are of great interest because of their ability to interact with atoms, ions, and molecules not only at their surfaces, but also throughout the bulk of the material [55–57]. Applications of porous materials mainly involve ion exchange, adsorption (for separation), and catalysis [57, 58]. On the basis of the multi-channel capillary pressures of PHOMs at oil/air and oil/water interfaces, PHOMs can spontaneously regulate using applied suction power [42]. It can prevent air and water permeating into the PHOMs to absorb floating oil. When the oil flows along the pipes to the collecting tank, PHOMs can regulate the oil present [42].

**Fig. 1:**
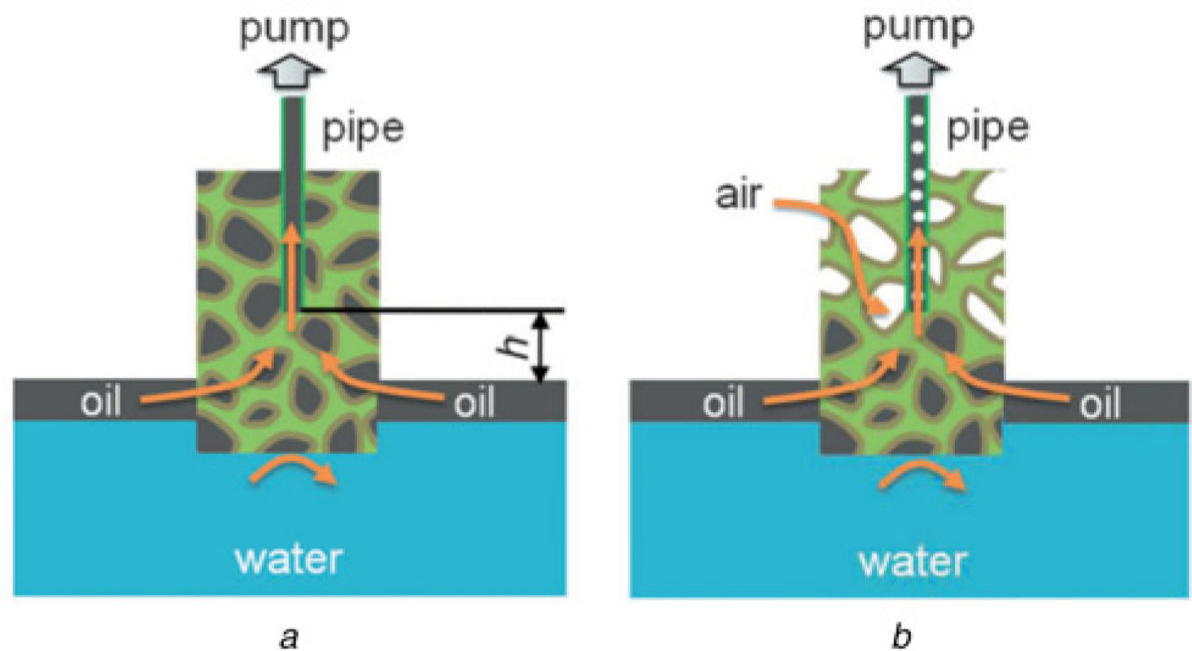
Capillary pressure at the oil/air interface transportation mechanisms with the HPS fully filled with oil, and with an air channel created in the HPS, respectively [42]. Reprinted with permission from Ref. [35]. Copyright 2014 John Wiley and Sons *a* water *b* oil

For example, Yu *et al*. reported the continuous collection of oil spills in situ from the water surface with high speed and efficiency [42]. This self-controlled oil collection system could collect oil spills easier and faster using a smaller amount of PHOM material. HPSs, which were obtained by modifying commercially available PSs with a hydrophobic silicon dioxide nanoparticle/polydimethylsiloxane coating through a facial dip coating method, were chosen as typical PHOMs. Capillary action at the oil/air and oil/water interfaces of HPS mainly contributed to the self-regulating properties of the system. As shown in Fig. 1, first the capillary pressure at the oil/air interface made the floating oil to be absorbed throughout the macropores of the HPS. However, after imposing a suction force to the HPS, because of the inverse relationship between the capillary pressure and the radius of curvature of the interfaces, the capillary pressure at the oil/air and oil/water interfaces increased subsequently due to the decrease of curvature radius of the interfaces to reach a new equilibrium with decreased pressure at the nozzle of the pipe. It would break the equilibrium between the pressure at oil surface and at the nozzle of the pipe, then the oil penetrated into the HPS through its surface. Then, the flow rate of oil decreased because the floating oil on the water surface vanished, which further caused the pressure at the pipe nozzle decreased. Then, the pressure at the oil/air interface was not able to keep such suction force that the oil above the pipe nozzle was pumped further away until an air channel formed in the HPS. Furthermore, this technique also could be applied to the separation of the organic-water phase, or kitchen waste oil material, or chemical production in the collection tank.

**Fig. 2:**
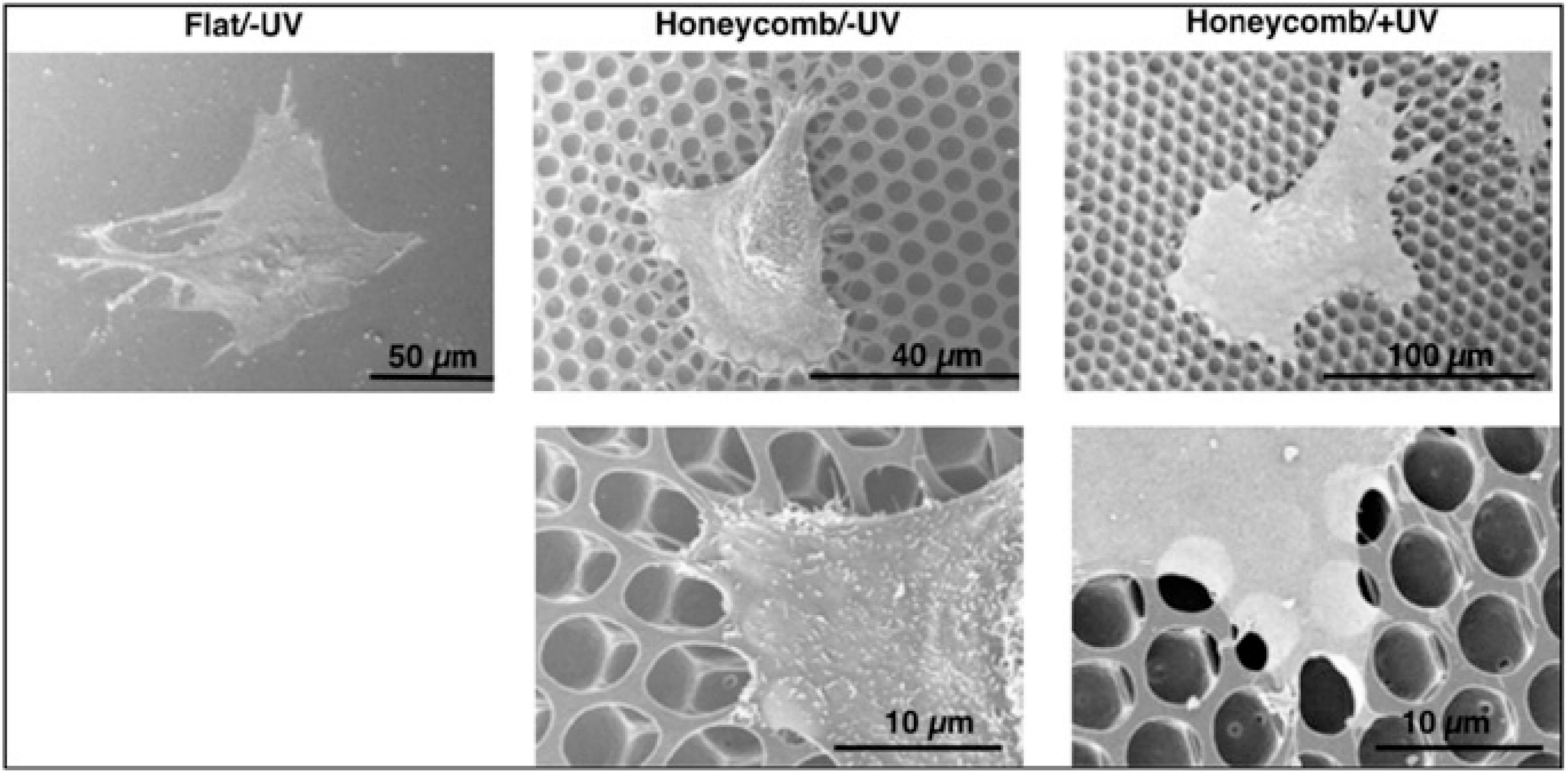
Scanning electron microscopy (SEM) images of fibroblasts on a flat substrate, a non-cross-linked honeycomb substrate, and a photo-cross-linked honeycomb substrate [40]. Reprinted from Ref. [33]. Copyright 2013 American Chemical Society

Kawano *et al*. [40] prepared honeycomb scaffolds for cell culturing by using ‘breath figure’ method, and they found that photo-cross-linking poly (1,2-butadiene) (PB) honeycomb cell culture substrates can modulate their deformation behaviour. They demonstrated that the regulation on a scale of several micrometres could affect the cell morphology and focal adhesion formation. In the cross-linked honeycomb scaffold, where the cells adhered tightly, as shown in Fig. 2, and did not have any deformation. On the contrary, the honeycomb structure was deformed and elongated against the cell edges in the non-cross-linked honeycomb scaffold. The microtography effect is one of the important factors for this regulation of honeycomb scaffolds, because cells in vivo usually grow on various regulated surroundings provided by other cells and extracellular matrices. Furthermore, the topological regulation of the cells is a biologically non-invasive strategy without the chemical factors. The microtopography effect of honeycomb scaffolds provides us a new method to understand the interaction between cells and material interface and scaffolds. On the basis of the multi-channel capillary pressures and cross-linked honeycomb scaffold, it can work together to construct an intelligent and efficient oil/water-controlled separation system.

## 3 Material based biological self-regulation systems

**Fig. 3:**
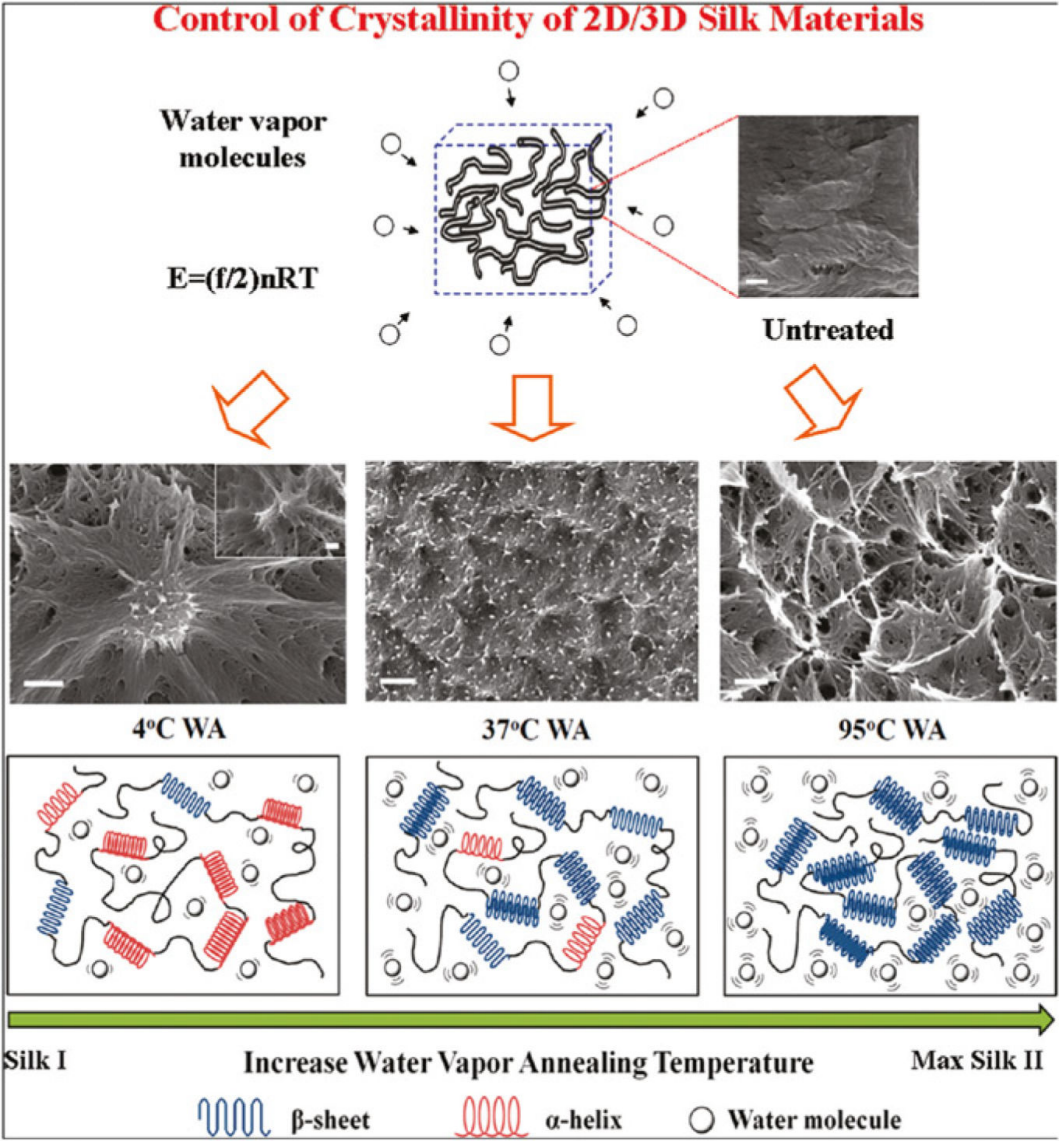
Mechanisms of temperature-controlled water annealing to silk fibroin proteins. Through the variation of vapour temperature, the structure of silk materials can be fully controlled from an R-helix-dominated silk I structure to the maximum β-sheet silk II structure. (Bar length in SEM images: 200 nm) [62]. Reprinted from Ref. [62]. Copyright 2011 American Chemical Society

**Fig. 4:**
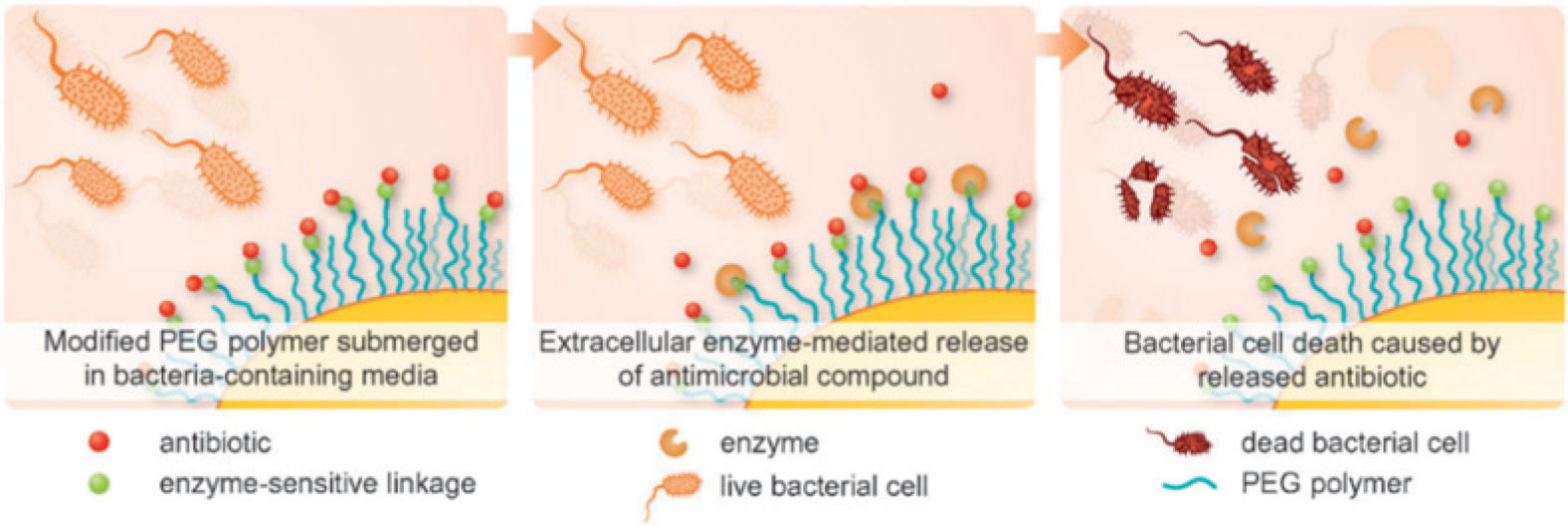
Concept of bacteria-trigged enzymatic release of antibiotics from chemically modified PEG [60]. Reprinted with permission from Ref. [60]. Copyright 2014 John Wiley and Sons

Self-adjusting sterilisation system based on the principle that system could release fungicide to disinfect due to their responses to biochemistry reactions caused by outside bacteria is a new research field in recent years [59–61]. Current research about this system is mainly focused on medical apparatuses and wound disinfection. For example, Nielsen *et al*. reported a self-adjusting poly(ethylene glycol) (PEG) for medical instrument disinfection [60]. As shown in Fig. 3, extracellular bacterial lipases are particularly abundant at sites of infection. When active or proactive compounds attached to polymeric surfaces using lipase-sensitive linkages such as fatty acid esters or anhydrides, fungicide may be released in response to infection. The key point of this system is that the precursor can be hydrolysed in bacterial media by extracellular lipases in the presence of a cleavable anhydride bond. When the accumulation of bacteria on the surface at the stage of early biofilm formation, the lipase is eventually provided and many compounds with a diverse range of biological activities such as compounds that will either stimulate or block quorum sensing (QB), may in principle be released to sensitise bacteria to the action of the immune system and antibiotics subsequently. This self-regulating system not only provides the basis for the development of device relevant polymeric materials, but also solves some of the key challenges for the treatment of bacterial infectious disease including both the mode of delivery, dosage of antibiotics at the site of infection, and development of antibiotic resistance (Fig. 4).

**Fig. 5:**
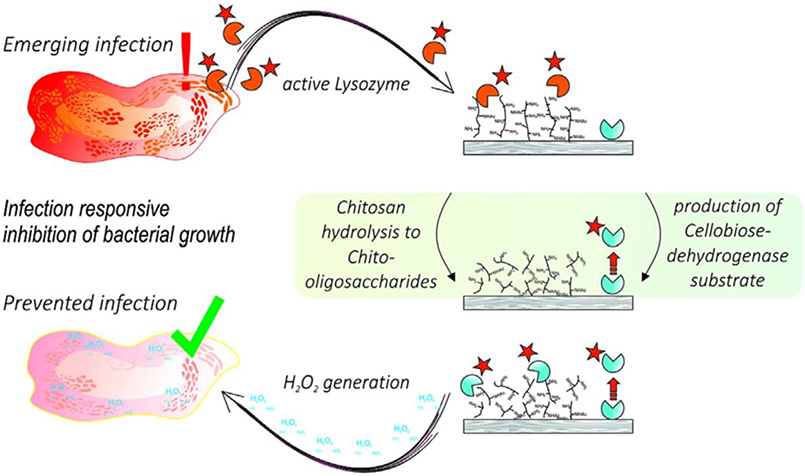
General scheme of the mode of action of the intelligent antimicrobial system. N - acetyl chitosan (NAc - CTS) is hydrolysed to COS in the presence of lysozyme. The produced COSs act as substrate for CDH that generates the antimicrobial agent H_2_O_2_ [63]. Reprinted with permission from Ref. [63]. Copyright 2017 John Wiley and Sons

The treatment of wound infection is one of the most serious threat in health care due to the increasing number of bacterial resistances and the difficulty of timely infection detection. For example, Tegl *et al*. reported a smart antimicrobial system that is activated in the case of infection based on elevated lysozyme activities [63]. First, *N*-acetyl chitosan (degree of *N*-acetylation: 40%) was synthesised and hydrolysis by lysozyme in artificial wound fluid to prepare *N*-acetylated chito-oligosaccharides (COSs) with a degree of polymerisation of 2–5 units. Then, the COS were used as substrate for cellobiose dehydrogenase (CDH) to produce antimicrobial hydrogen peroxide (H_2_O_2_). This approach represents the first self-regulating system for the infection responsive inhibition of bacterial growth in response to lysozyme as infection biomarker. The results of simulated experiments in artificial wound showed that the system had a good work ability to generate up to 1 mM H_2_O_2_ at physiological conditions. This system by combining lysozyme responsive *N*-acetyl chitosan and CDH could facilitate or be used for the potential treatment of bacterial wound. Potentially, the above self-regulated antibacterial model could be a role as an important part in intelligent medical treatment engineering (Fig. 5).

## 4 Self-regulated polymerisation

**Fig. 6:**
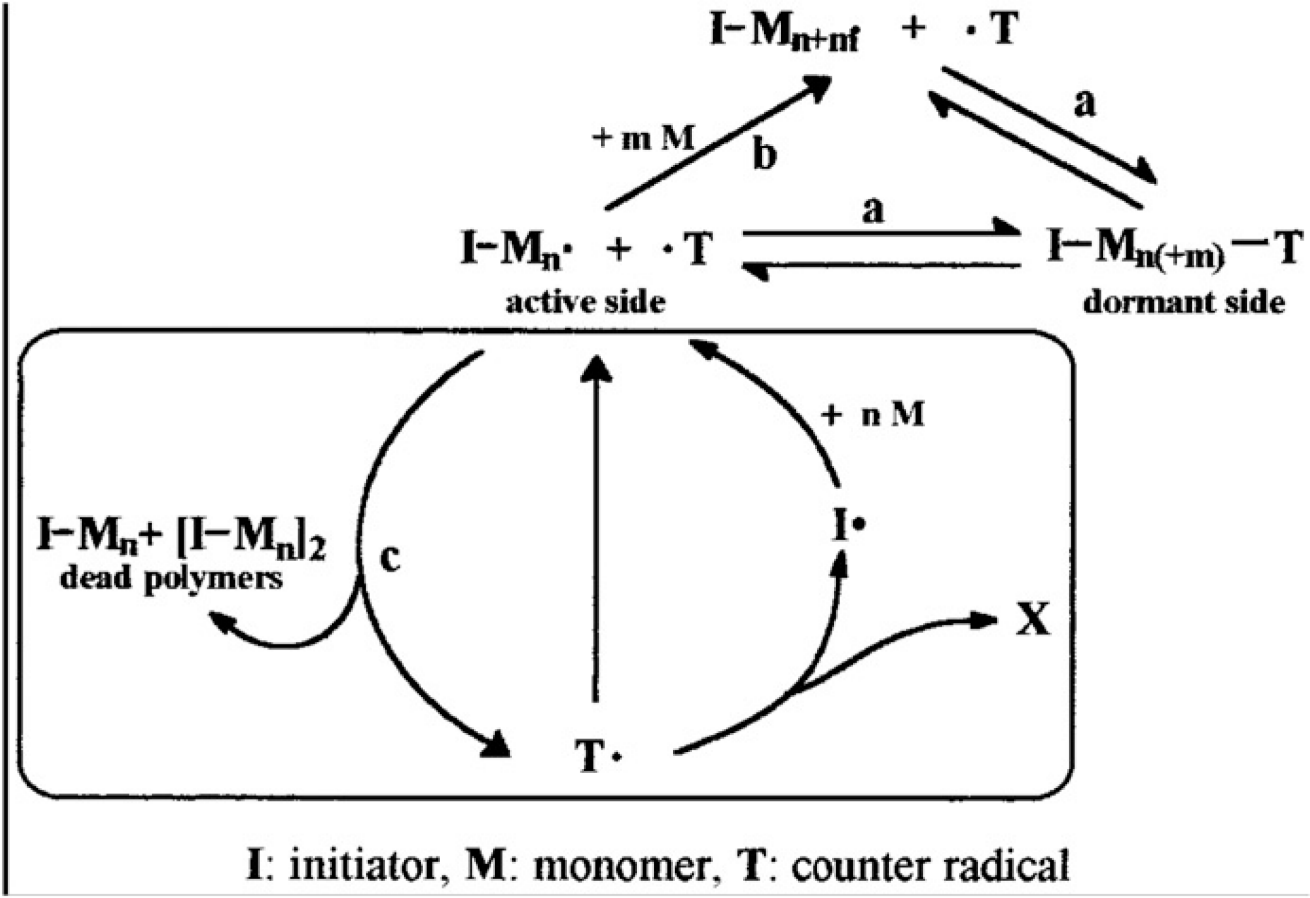
Controlled radical polymerisation in the presence of triazolinyl 4 [68]. Reprinted from Ref. [68]. Copyright 1998 American Chemical Society

Recently, the development of controlled radical polymerisation has attracted much attention [64–67]. For example, Steenbock *et al*.[68] reported the decomposition of stable free radicals as self-regulation’ in controlled radical polymerisation of styrene. The polymerisation reaction stops could be catalysed by irreversible side reactions of the free radicals, the amount of dead polymer chains, and, consequently, the concentration of stable free radicals increases during the polymerisation reaction. They found that triazolinyl four radicals could reduce its own free concentration in solution, and therefore to establish a self-regulating reaction cycle. They provided two possible reasons for radical four to prevent the increase of free radical concentration in solution. First, it slows the thermal reinitiation of the monomer and second it can decompose to new initiating radicals to control its free concentration in solution. The triazolinyl four controlled polymerisation of styrene is a self-regulating mechanism instead of the slow thermal initiation of styrene (Fig. 6).

Xu *et al*. reported the fluorescence in a responsive polymer system was autonomously regulated based on the reversible formation of dynamic covalent bonds by utilising a chemical oscillating reaction as a periodical driving source [69]. Yao *et al*. found that the conformation of polyaniline (PANI) molecules changed from a compacted coil to an expanded coil when they prepared pure PANI films with different molecular chain packing states by simply tuning the *m*-cresol content in the solvent [70]. This phenomenon is due to enhancement of the delocalisation of *p*-polarons along the polymer chain caused by chemical interactions between PANI and *m*-cresol. Furthermore, with increasing the *m*-cresol content, the ordered regions in PANI molecular structures were enhanced because *m*-cresol could decrease the hopping barrier and increase the carrier mobility. Especially, the electrical conductivity increased from 4.7 to 220 S cm^−1^ when the *m*-cresol content changed from 0 to 100%.

Hu *et al*. [62] used a simple and effective method to obtain refined control of the molecular structure of porous silk biomaterials through physical temperature-controlled water vapour annealing (TCWVA). The crystallinity of silk materials can be controlled from a low content using conditions at 4°C (R-helix-dominated silk I structure), to the highest content of ∼60% crystallinity at 100°C (*β*-sheet dominated silk II structure). This new approach to control crystallisation also provides an entirely new green approach without using organic solvents. The method using water to control chain interactions described here may be suitable for silk proteins and many other structural proteins. Controlled radical polymerisation and TCWVA method can be applied to intelligent synthesis, both of them provide a self-regulating reaction synthetic method for polymer materials with special properties.

## 5 Photoelectric self-regulation

**Fig. 7:**
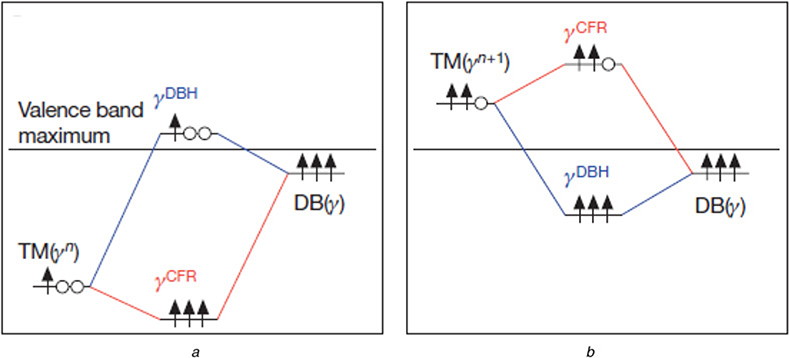
Energy-level diagrams for TM–host interaction *a* System charge, *q* *b* System charge. In each panel, the free-TM-atom orbital with irreducible representation (left-hand side) interacts with a host dangling bond level with the same representation (right-hand side), forming two hybrid levels (centre), namely the CFR level CFR and a DBH level DBH. The arrows indicate spin-up electrons occupying the level, and the circles indicate unoccupied states [72]. Reprinted by permission from Macmillan Publishers Ltd: Nature [59], copyright (2008)

Classic inorganic chemistry of TM coordination compounds and semiconductors tacitly assumes that the total charge of the compound will be mainly accomplished by the changes in the charge of TM ions [71, 72]. However, in some cases, this change of charge is considered to be physical entities or capable of physical interactions. Raebiger *et al*. [72] studied multiple-charge configurations of isolated TM atoms in representative host materials including the archetypal ionic magnesium oxide, and covalent compounds gallium arsenide and an intermediate case involving d electrons-Cu_2_O. Fig. 7 shows the negative charge feedback in their work. Here, *n* electrons occupied the isolated TM (*d*) orbits to split into crystal field levels. If the system with *q* charge in Fig. 7a is doped with an electron, the system charge becomes to *q*−*1* in Fig. 7b. Then, the level occupancy of the TM-induced hybrid states increases, the relative weight of the bonding levels shifts toward the ligands and such charge doping consequently causes the bonding and anti-bonding hybrid levels to shift up in energy by means of a negative feedback. Typically, when the energy of the TM(*γ*) level is lower than that of the DB(*γ*) level, as shown in Fig. 7a, the bonding level occurs as a crystal field resonance (CFR) with a strong TM character and the anti-bonding level occurs as a dangling bond hybrid (DBH) formed mostly of the ligand orbits. In addition, to counterbalance the increase in the anti-bonding gap-level charge, this negative feedback causes a reduction of TM charge in the bonding states. In other words, this self-regulated response could keep the net local charge at the TM site approximately constant. In this model, the change of charge in TM coordination compounds and semiconductors reflect the occupancy of the respective crystal field levels rather than the local charge at the TM site [60].

Zhou *et al*. reported the preparation of titanium dioxide (TiO_2_) composite nanofibres by solvent volatilisation during the electrospinning process. Their results showed that the surface area to volume of the obtained TiO_2_ nanofibres was enlarged with the increased weight fraction of tetrahydrofuran in the hybrid solvent. The structure and the surface area to volume ratio of the fibres could be adjusted by regulating the weight ratio of the solvent in the mixture and even by calcinations [73]. Lu *et al*. found that tunable emission from green to red and the inverse tuning from red to green in *α*^′^_L_-(Ca,Sr)_2_SiO_4_:Eu^2+^phosphors were demonstrated magically by varying the incorporation content of Eu^2+^ and Sr^2+^ ions [74]. The change of emitting centres from ten- to eight-coordinated sites caused by the increase of Eu^2+^ incorporation concentration was the reason for the red-shifting from 546 to 638 nm in *α*^′^_L_-(Ca,Sr)_2_SiO_4_:Eu^2+^ phosphors. Moreover, the lattice expansion induced crystal field splitting decrease resulted in the blue-shifting from 638 to 540 nm in *α*^′^_L_Ca_1.6−_ ySr_y_SiO_4_:Eu^2+^ phosphors alone with the change of the Sr^2+^ incorporation content. Self-regulated occupancy of the charge in TM coordination compounds, self-regulated change of emitting centres, and self-regulated synthesis of photoelectric materials will greatly promote the rapid application of intelligent semiconductor devices.

## 6 Self-regulated mechanochemical system

**Fig. 8:**
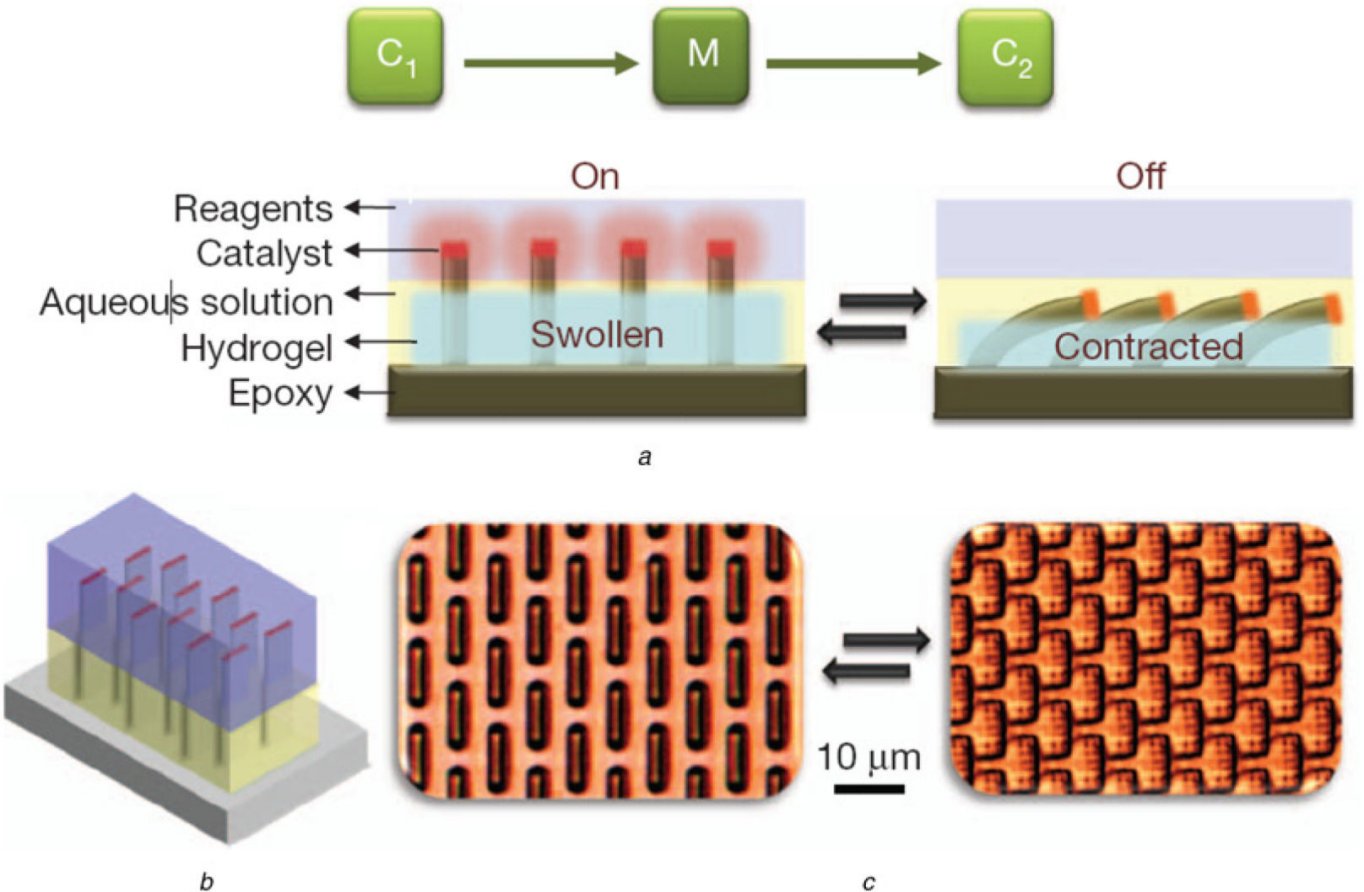
General design of SMARTS *a* Cross-section schematic representation *b* Three-dimensional schematic representation [25]. Reprinted by permission from Macmillan Publishers Ltd: Nature [25], copyright (2012)

Biochemical reactions involved with SMARTS can control innumerably sophisticated, environmentally friendly, enzymatic, or other biological activities useful for self-regulating medical implants such as biomolecule detection, separation, and signal amplification [25]. He *et al*. [25] demonstrated precise control of biochemical signal transduction using a model system composed of reconfiguration of the gel to microstructure tips, catalysing the oxidation of luciferin in an oscillatory fashion. The self-regulated reaction in this work is SMARTS, which can be tailored to a broad range of coupled chemomechanical and mechanochemical events. They applied fluoresce into the microstructure tips and observed the on/off states of fluoresce in quenching by potassium iodide in the nutrient layer. The SMARTS device could be compatible with delicate biological constraints and capable of accommodating enzymatic reactions for signal transduction. The potential variety of switchable biochemical reactions that could be accommodated by this C1→M→C2 cascade (as shown in Fig. 8) is complemented by the customisability of the hydrogel response, which can, in turn, be tailored to a wide range of stimuli such as pH, heat, light, glucose, or other metabolic compounds, as well as the vast varieties of outputs such as gas generation, colour change, DNA polymerisation, and proteolysis, thus improving the combinatorial diversity of coupled effects. In the future work, they anticipated using of SMARTS to maintain a stable temperature could be used in autonomous self-sustained thermostats with applications ranging from medical implants.

Mzyk *et al*. [75] reported chemo-mechano-regulation of endothelial cell response based on polyelectrolyte multilayer film modification. In this system, multilayer polyelectrolyte films (PEMs) are able to simulate the structure and functions of the extracellular matrix. The bioactive PEM was investigated as a supportive system for efficient endothelialisation of cardiovascular implants. The results showed that the PEM rigidity was regulated by the cross-linking chemistry as well as nanoparticle incorporation. Bionic biological monitoring and sensing is an important node of intelligent engineering, and the self-regulating system with biological detection, separation, and signal amplification can even be expected to use for autonomous self-sustained thermostats. This makes the intelligent materials more broad application prospect in the field of intelligent engineering.

## 7 Self-regulated energy materials

Recently, lithium-ion batteries (LIBs) and sodium-ion batteries (SIBs) are considered the high-efficiency and environment-friendly energy storage systems, due to their outstanding features which include high-energy density, high-power density, and high-cycle life [76–78]. Many alternative power materials have been explored as potential anodes for LIBs and SIBs such as carbon materials, metal oxides, TM chalcogenides, and alloy materials [79–81]. Self-regulated energy materials with hollow structure and carbon modification have been used to improve their electrochemical performances.

Li *et al*. reported mesoporous CoS@alveolus-like carbon yolk–shell microsphere for alkali cations storage. In this unique yolk–shell microsphere, the alveolus-like carbon shells provide a highly self-regulated void space to guarantee the integrity of mesoporous CoS during the below cycling process:

$$\text{CoS+2Li/Na}\to {\text{Co+Li}}_{\text{2}}{\text{S/Na}}_{\text{2}}\text{S}$$

**Fig. 9:**
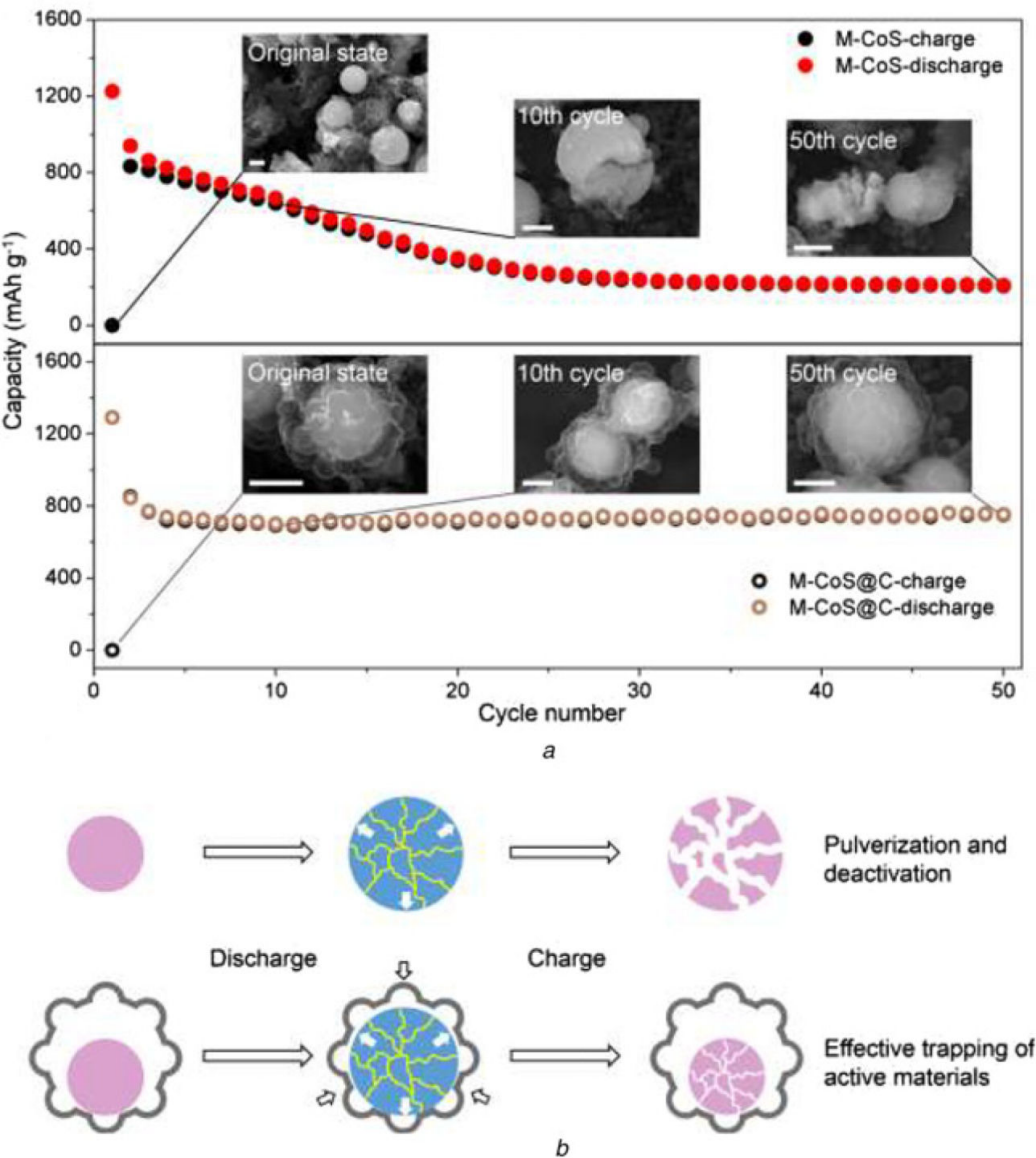
Morphological changes of the M-CoS and yolk–shell M-CoS@C during the cycling process *a* SEM images of the M-CoS and yolk–shell M-CoS@C at 0, 10th, and 50th cycles (full charge state), and their corresponding cycling performances at 1 A g^−1^. Scale bar, 1 μm *b* Schematic representation of the morphological changes during the discharge/charge process. Reprinted from [81]. Copyright 2017 Elsevier

Without self-regulated structure, the volume expansion of CoS will not be controlled in process of discharging and result in the fracture of the particle. During the charging process, the fragments of CoS materials shrink and separate from each other, causing the electrode to be crushed and inactivated. Once constructing the unique self-regulated structure of the yolk–shell, similar to the expansion of alveoli in the process of respiration, the carbon shell with alveolar function limited the volume expansion of sulphide cobalt particles during the discharge process. These phenomena show that the unique alveolar carbon shell can effectively bear the huge volume changes of the active electrode material and greatly improve the circulation stability. It is similar to the important role of temperature self-sustaining system in intelligent engineering, self-regulated energy materials could be anticipated to supply a constant energy in the complex and variable environment (Fig. 9).

## 8 Conclusions and perspectives

Self-regulation is proved to widely exist in many well-designed materials/environment systems. For example, porous materials can self-regulate their structure such as pore size and density, according to the changes in their environment. Biological materials need to adapt the living body through self-regulation of biological reactions. The self-regulation of stable free radicals in controlled radical polymerisation could be used to control the polymerisation processes. The photoelectric materials are sensitive to the environment due to a series of self-regulation processes. Biochemical reactions involved with SMARTS can control innumerable sophisticated, environmentally friendly, enzymatic, or other biological activities. On the basis of the theory of self-regulation, the self-regulated materials are in prospect for the application not only in self-regulation industry, but also in the biological field such as emotional self-regulation and homeostasis of a living body, and self-control in sociology/psychology and self-learning of animals and human beings. Even the assembly of parts with different self-regulating characteristics will form a complex intelligent system, which will play a larger role in the future intelligent society.

For future development, the knowledge bases regarding for examples, designing the self-regulation system and their interactions throughout the medical; mechanical properties, energy control, and their influences on the interface are still incomplete. These materials science aspects still need further investigation. Meanwhile, the characterisation and designing strategy vary from system to system, and standards need to be established to ensure better quality control. Furthermore, relative theoretical instruction tailing for new systems need to be stored. In summary, the outlook on discovering the self-regulated system and utilising self-regulation method on material and medical appliances, formed by current methods, as a smart interface to aid the functional process, is favourable. Future developments that revolve around process/material control in order to predetermine the precise regulation and exact function will assure agreeable future results. In fact, in addition to materials, self-regulation could be found in almost all of the living bodies. The breakthroughs in self-regulated materials and bio-self-regulation are expected to archive in the near future. However, the basic theory of self-regulation is still not impeccable.

## References

[1] Verma A, Uzun O, Hu Y, : ‘Surface-structure-regulated cell-membrane penetration by monolayer-protected nanoparticles’, Nat. Mater, 2008, 7, p. 588 18500347 10.1038/nmat2202 PMC2684029

[2] Chaudhuri O, Gu L, Klumpers D, : ‘Hydrogels with tunable stress relaxation regulate stem cell fate and activity’, Nat. Mater., 2015, 15, pp. 326–334 26618884 10.1038/nmat4489 PMC4767627

[3] Schoen I, Pruitt BL, Vogel V: ‘The yin-yang of rigidity sensing: how forces and mechanical properties regulate the cellular response to materials’, Annu. Rev. Mater. Res, 2013, 43, p. 589

[4] Montfort J-P, Indirli G, Georgieva D, : ‘Nanomaterials under REACH: Legal aspects: Unless and until REACH is adapted to more specifically regulate nanomaterials, is there scope for national measures to regulate these materials?’, Eur. J. Risk Regul, 2010, 1, p. 51

[5] Li S, Du L, Yuen G, : ‘Supplemental Materials for “Distinct Ceramide Synthases Regulate Polarized Growth in the Filamentous Fungus Aspergillus nidulans D”‘, vol. 2 (Liangcheng Du Publications, 2006) 10.1091/mbc.E05-06-0533 PMC1382311 16394102

[6] Nagai H, Irie T, Takahashi J, : ‘Flexible manipulation of microfluids using optically regulated adsorption/desorption of hydrophobic materials’, Biosens. Bioelectron, 2007, 22, p. 1968 17027249 10.1016/j.bios.2006.08.037

[7] Sarbassov DD, Guertin DA, Ali SM, : ‘Phosphorylation and regulation of Akt/PKB by the rictor-mTOR complex’, Science, 2005, 307, p. 1098 15718470 10.1126/science.1106148

[8] Fukuda R: ‘Metabolism of hydrophobic carbon sources and regulation of it in n-alkane-assimilating yeast Yarrowia lipolytica’, Biosci. Biotechnol. Biochem, 2013, 77, p. 1149 23748781 10.1271/bbb.130164

[9] Bayascas JR, Alessi DR: ‘Regulation of Akt/PKB Ser473 phosphorylation’, Mol. Cell, 2005, 18, p. 143 15837416 10.1016/j.molcel.2005.03.020

[10] Bai W, Sheng Q, Ma X, : ‘Synthesis of silver nanoparticles based on hydrophobic interface regulation and its application of electrochemical catalysis’, ACS Sustain. Chem. Eng, 2015, 3, p. 1600

[11] Sanchez S, Chávez A, Forero A, : ‘Carbon source regulation of antibiotic production’, J. Antibiot, 2010, 63, p. 442 10.1038/ja.2010.78 20664603

[12] Liu G, Chater KF, Chandra G, : ‘Molecular regulation of antibiotic biosynthesis in Streptomyces’, Microbiol. Mol. Biol. Rev, 2013, 77, p. 112 23471619 10.1128/MMBR.00054-12 PMC3591988

[13] McCallum N, Berger-Bächi B, Senn MM: ‘Regulation of antibiotic resistance in Staphylococcus aureus’, Int. J. Med. Microbiol, 2010, 300, p. 118 19800843 10.1016/j.ijmm.2009.08.015

[14] Bibb M, Hesketh A: ‘Analyzing the regulation of antibiotic production in streptomycetes’, Methods Enzymol, 2009, 458, p. 93 19374980 10.1016/S0076-6879(09)04804-6

[15] Cundliffe E: ‘Antibiotic production by actinomycetes: the Janus faces of regulation’, J. Ind. Microbiol. Biotechnol, 2006, 33, p. 500 16463161 10.1007/s10295-006-0083-6

[16] Hibi Y, Ouchi M, Sawamoto M: ‘Sequence-regulated radical polymerization with a metal-templated monomer: repetitive aba sequence by double cyclopolymerization’, Angew. Chem, 2011, 123, p. 7572 10.1002/anie.201103007 21717555

[17] Satoh K, Ozawa S, Mizutani M, : ‘Sequence-regulated vinyl copolymers by metal-catalysed step-growth radical polymerization’, Nat. Commun, 2010, 1, p. 6 20975670 10.1038/ncomms1004

[18] Treat NJ, Fors BP, Kramer JW, : ‘Controlled radical polymerization of acrylates regulated by visible light’, ACS Macro Lett, 2014, 3, p. 580 10.1021/mz500242a 35590731

[19] Nakatani K, Ogura Y, Koda Y, : ‘Sequence-regulated copolymers via tandem catalysis of living radical polymerization and in situ transesterification’, J. Am. Chem. Soc, 2012, 134, p. 4373 22296320 10.1021/ja211436n

[20] De P, Li M, Gondi SR, : ‘Temperature-regulated activity of responsive polymer–protein conjugates prepared by grafting-from via RAFT polymerization’, J. Am. Chem. Soc, 2008, 130, p. 11288 18665597 10.1021/ja804495v

[21] Sun Y, Gao S, Xie Y: ‘Atomically-thick two-dimensional crystals: electronic structure regulation and energy device construction’, Chem. Soc. Rev, 2014, 43, p. 530 24122032 10.1039/c3cs60231a

[22] Piva PG, DiLabio GA, Pitters JL, : ‘Field regulation of single-molecule conductivity by a charged surface atom’, Nature, 2005, 435, p. 658 15931218 10.1038/nature03563

[23] Zheng R, Gao J, Wang J, : ‘Reversible temperature regulation of electrical and thermal conductivity using liquid–solid phase transitions’, Nat. Commun, 2011, 2, p. 289 21505445 10.1038/ncomms1288 PMC3104514

[24] Shi J-N, Ger M-D, Liu Y-M, : ‘Improving the thermal conductivity and shape-stabilization of phase change materials using nanographite additives’, Carbon, 2013, 51, p. 365

[25] He X, Aizenberg M, Kuksenok O, : ‘Synthetic homeostatic materials with chemo-mechano-chemical self-regulation’, Nature, 2012, 487, p. 214 22785318 10.1038/nature11223

[26] Persat A, Inclan YF, Engel JN, : ‘Type IV pili mechanochemically regulate virulence factors in Pseudomonas aeruginosa’, Proc. Natl. Acad. Sci, 2015, 112, p. 7563 26041805 10.1073/pnas.1502025112 PMC4475988

[27] Koride S, He L, Xiong L-P, : ‘Mechanochemical regulation of oscillatory follicle cell dynamics in the developing Drosophila egg chamber’, Mole. Biol. Cell, 2014, 25, p. 3709 10.1091/mbc.E14-04-0875 PMC4230628 24943847

[28] Hu L, Papoian GA: ‘Mechano-chemical feedbacks regulate actin mesh growth in lamellipodial protrusions’, Biophys. J, 2010, 98, p. 1375 20409456 10.1016/j.bpj.2009.11.054 PMC2856144

[29] Astrof NS, Salas A, Shimaoka M, : ‘Importance of force linkage in mechanochemistry of adhesion receptors’, Biochemistry, 2006, 45, p. 15020 17154539 10.1021/bi061566o PMC1766327

[30] Chen J, Le S, Basu A, : ‘Mechanochemical regulations of RPA’s binding to ssDNA’, Sci. Rep, 2015, 5, 9296–9303 25787788 10.1038/srep09296 PMC4365408

[31] Herschlag G, Garcia GJ, Button B, : ‘A mechanochemical model for auto-regulation of lung airway surface layer volume’, J. Theor. Biol, 2013, 325, p. 42 23415939 10.1016/j.jtbi.2013.01.023 PMC3631568

[32] Wang C, Wu Y, Li Y, : ‘Flame-retardant rigid polyurethane foam with a phosphorus-nitrogen single intumescent flame retardant’, Polym. Adv. Technol, 2018, 29, 668–676

[33] Liu Z, Huang Z, Cheng F, : ‘Efficient dual-site carbon monoxide electro-catalysts via interfacial nano-engineering’, Sci. Rep, 2016, 6, 33127–33137 27650532 10.1038/srep33127 PMC5030650

[34] Huang J, Cao Y, Huang Z, : ‘Comparatively thermal and crystalline study of poly(methyl-methacrylate)/polyacrylonitrile hybrids: core–shell hollow fibers, porous fibers, and thin films’, Macromol. Mater. Eng, 2016, 301, 1327–1336 29104455 10.1002/mame.201600172 PMC5669389

[35] Zhang L, Yu W, Han C, : ‘Large scaled synthesis of heterostructured electrospun TiO_2_/SnO_2_ nanofibers with an enhanced photocatalytic activity’, J. Electrochem. Soc, 2017, 164, p. H651

[36] Zhang K, Li G-H, Feng L-M, : ‘Ultralow percolation threshold and enhanced electromagnetic interference shielding in poly(l-lactide)/multi-walled carbon nanotubes nanocomposites with electrically conductive segregated networks’, J. Mater. Chem. C, 2017, 5, 9359–9369

[37] Sun Z, Zhang L, Dang F, : ‘Experimental and simulation-based understanding of morphology controlled barium titanate nanoparticles under co-adsorption of surfactants’, CrystEngComm, 2017, 19, (24), 3288–3298

[38] Li Y, Wu X, Song J, : ‘Reparation of recycled acrylonitrile-butadiene-styrene by pyromellitic dianhydride: Reparation performance evaluation and property analysis’, Polymer, 2017, 124, 41–47

[39] Liao P-Q, Zhu A-X, Zhang W-X, : ‘Self-catalysed aerobic oxidization of organic linker in porous crystal for on-demand regulation of sorption behaviours’, Nat. Commun, 2015, 6, p. 6350 25702689 10.1038/ncomms7350

[40] Kawano T, Nakamichi Y, Fujinami S, : ‘Mechanical regulation of cellular adhesion onto honeycomb-patterned porous scaffolds by altering the elasticity of material surfaces’, Biomacromolecules, 2013, 14, p. 1208 23510479 10.1021/bm400202d

[41] Ding K, Corma A, Maciá-Agulló JA, : ‘Constructing hierarchical porous zeolites via kinetic regulation’, J. Am. Chem. Soc, 2015, 137, p. 11238 26322625 10.1021/jacs.5b06791

[42] Ge J, Ye YD, Yao HB, : ‘Pumping through porous hydrophobic/oleophilic materials: an alternative technology for oil spill remediation’, Angew. Chem, 2014, 126, p. 3686 10.1002/anie.201310151 24591265

[43] Yoshii R, Tanaka K, Chujo Y: ‘Conjugated polymers based on tautomeric units: regulation of main-chain conjugation and expression of aggregation induced emission property via boron-complexation’, Macromolecules, 2014, 47, p. 2268

[44] Qi F, Wang Y, Ma T, : ‘Electrical regulation of olfactory ensheathing cells using conductive polypyrrole/chitosan polymers’, Biomaterials, 2013, 34, p. 1799 23228424 10.1016/j.biomaterials.2012.11.042

[45] Patel S, Tsang J, Harbers GM, : ‘Regulation of endothelial cell function by GRGDSP peptide grafted on interpenetrating polymers’, J. Biomed. Mater. Res. A, 2007, 83, p. 423 17455217 10.1002/jbm.a.31320

[46] Wen L, Hou X, Tian Y, : ‘Bioinspired smart gating of nanochannels toward photoelectric-conversion systems’, Adv. Mater, 2010, 22, p. 1021 20217833 10.1002/adma.200903161

[47] Sinha P, Kriegner CJ, Schew WA, : ‘Regulatory policy governing cadmium-telluride photovoltaics: A case study contrasting life cycle management with the precautionary principle’, Energy Policy, 2008, 36, p. 381

[48] Mao Z, Lu Z, Chen J, : ‘Tunable luminescent Eu^2+^-doped dicalcium silicate polymorphs regulated by crystal engineering’, J. Mater. Chem. C, 2015, 3, p. 9454

[49] Sonenberg N, Hinnebusch AG: ‘Regulation of translation initiation in eukaryotes: mechanisms and biological targets’, Cell, 2009, 136, p. 731 19239892 10.1016/j.cell.2009.01.042 PMC3610329

[50] Blair C, Diamond A: ‘Biological processes in prevention and intervention: The promotion of self-regulation as a means of preventing school failure’, Dev. Psychopathol, 2008, 20, p. 899 18606037 10.1017/S0954579408000436 PMC2593474

[51] Hare TA, Tottenham N, Galvan A, : ‘Biological substrates of emotional reactivity and regulation in adolescence during an emotional go-nogo task’, Biol. Psychiatry, 2008, 63, p. 927 18452757 10.1016/j.biopsych.2008.03.015015 PMC2664095

[52] Foyer CH, Noctor G: ‘Redox regulation in photosynthetic organisms: signaling, acclimation, and practical implications’, Antioxid. Redox Signal, 2009, 11, p. 861 19239350 10.1089/ars.2008.2177

[53] Archambault V, Glover DM: ‘Polo-like kinases: conservation and divergence in their functions and regulation’, Nat. Rev. Mol. Cell Biol, 2009, 10, p. 265 19305416 10.1038/nrm2653

[54] Nieves-Cordones M, Alemán F, Martínez V, : ‘K^+^ uptake in plant roots. The systems involved, their regulation and parallels in other organisms’, J. Plant Physiol, 2014, 171, p. 688 24810767 10.1016/j.jplph.2013.09.021

[55] Lu AH, Schüth F: ‘Nanocasting: a versatile strategy for creating nanostructured porous materials’, Adv. Mater, 2006, 18, p. 1793

[56] Kitagawa S: ‘Future porous materials’, Acc. Chem. Res, 2017, 50, p. 514 28945427 10.1021/acs.accounts.6b00500

[57] Davis ME: ‘Ordered porous materials for emerging applications’, Nature, 2002, 417, p. 813 12075343 10.1038/nature00785

[58] Wang Z, Colorad HA, Guo Z-H, : ‘Effective functionalization of carbon nanotubes for bisphenol F epoxy matrix composites’, Mater. Res., 2012, 15, p. 510

[59] Paranjpe A, De Gregorio C, Gonzalez AM, : ‘Efficacy of the self-adjusting file system on cleaning and shaping oval canals: a microbiological and microscopic evaluation’, J. Endod, 2012, 38, p. 226 22244642 10.1016/j.joen.2011.10.014

[60] Komnatnyy VV, Chiang WC, Tolker-Nielsen T, : ‘Bacteria-triggered release of antimicrobial agents’, Angew. Chem. Int. Ed, 2014, 53, p. 439 10.1002/anie.201307975 24288347

[61] Wang Z, Sun C, Vegesna G, : ‘Glycosylated aniline polymer sensor: amine to imine conversion on protein-carbohydrate binding’, Biosens. Bioelectron, 2013, 46, 183–189 23563436 10.1016/j.bios.2013.02.030 PMC3947566

[62] Hu X, Shmelev K, Sun L, : ‘Regulation of silk material structure by temperature-controlled water vapor annealing’, Biomacromolecules, 2011, 12, p. 1686 21425769 10.1021/bm200062a PMC3090511

[63] Öhlknecht C, Tegl G, Beer B, : ‘Cellobiose dehydrogenase and chitosan-based lysozyme responsive materials for antimicrobial wound treatment’, Biotechnol. Bioeng, 2017, 114, p. 416 27500401 10.1002/bit.26070

[64] Aqeel SM, Wang Z, Than L, : ‘Poly(vinylidene fluoride)/poly (acrylonitrile)–based superior hydrophobic piezoelectric solid derived by aligned carbon nanotubes in electrospinning: fabrication, phase conversion and surface energy’, RSC Adv, 2015, 5, p. 76383 26989486 10.1039/C5RA11584A PMC4792282

[65] Wang Z, Yang X, Wang Q, : ‘Epoxy resin nanocomposites reinforced with ionized liquid stabilized carbon nanotubes’, Int. J. Smart Nano Mater, 2011, 2, p. 176

[66] Wang Z, Zhu ZZ, Shi J, : ‘Electrocatalytic oxidation of formaldehyde on platinum well-dispersed into single-wall carbon nanotube/polyaniline composite film’, Appl. Surf. Sci, 2007, 253, p. 8811

[67] Wang Z, Lu M, Li H-L, : ‘SWNTs–polystyrene composites preparations and electrical properties research’, Mater. Chem. Phys, 2006, 100, p. 77

[68] Steenbock M, Klapper M, Müllen K, : ‘Decomposition of stable free radicals as “self-regulation” in controlled radical polymerization’, Macromolecules, 1998, 31, p. 5223

[69] Xu G, Zhou H, Li J, : ‘Autonomous fluorescence regulation in responsive polymer systems driven by a chemical oscillating reaction’, Polym. Chem., 2016, 7, p. 3211

[70] Yao Q, Wang Q, Wang L, : ‘The synergic regulation of conductivity and Seebeck coefficient in pure polyaniline by chemically changing the ordered degree of molecular chains’, J. Mater. Chem. A, 2014, 2, p. 2634

[71] Goodenough J, Rivadulla F: ‘Bond-length fluctuations in transition-metal oxides’, Mod. Phys. Lett. B, 2005, 19, p. 1057

[72] Raebiger H, Lany S, Zunger A: ‘Charge self-regulation upon changing the oxidation state of transition metals in insulators’, Nature, 2008, 453, p. 763 18528391 10.1038/nature07009

[73] Zhou Z, Xiao W, Shi X, : ‘Pore volume and distribution regulation of highly nanoporous titanium dioxide nanofibers and their photovoltaic properties’, J. Colloid Interface Sci, 2017, 490, p. 74 27870962 10.1016/j.jcis.2016.11.035

[74] Lu Z, Mao Z, Chen J, : ‘Red/blue-shift dual-directional regulation of α′L-(Ca, Sr)2SiO4:Eu2+ phosphors resulting from the incorporation content of Eu2+/Sr2+ ions’, Dalton Trans, 2015, 44, p. 15620 26245750 10.1039/c5dt01690h

[75] Mzyk A, Lackner JM, Wilczek P, : ‘Polyelectrolyte multilayer film modification for chemo-mechano-regulation of endothelial cell response’, RSC Adv., 2016, 6, p. 8811

[76] Kang B, Ceder G: ‘Battery materials for ultrafast charging and discharging’, Nature, 2009, 458, p. 190 19279634 10.1038/nature07853

[77] Vogt LO, El Kazzi M, Jämstorp Berg E, : ‘Understanding the interaction of the carbonates and binder in na-ion batteries: a combined bulk and surface study’, Chem. Mater, 2015, 27, p. 1210

[78] Cao Y, Xiao L, Sushko ML, : ‘Sodium ion insertion in hollow carbon nanowires for battery applications’, Nano Lett, 2012, 12, p. 3783 22686335 10.1021/nl3016957

[79] Hasa I, Verrelli R, Hassoun J: ‘Transition metal oxide-carbon composites as conversion anodes for sodium-ion battery’, Electrochim. Acta, 2015, 173, p. 613

[80] Zhang K, Park M, Zhou L, : ‘Cobalt-doped FeS_2_ nanospheres with complete solid solubility as a high-performance anode material for sodium-ion batteries’, Angew. Chem. Int. Ed, 2016, 55, p. 12822 10.1002/anie.201607469 27624365

[81] Li Q, Li L, Owusu KA, : ‘Self-adaptive mesoporous CoS@alveolus-like carbon yolk-shell microsphere for alkali cations storage’, Nano Energy, 2017, 41, p. 109

